# Percutaneous full-endoscopic anterior transcorporeal cervical discectomy and channel repair: a technique note report

**DOI:** 10.1186/s12891-019-2659-0

**Published:** 2019-06-10

**Authors:** Qian Du, Lan-Qiong Lei, Guan-Ru Cao, Wei-Jun Kong, Jun Ao, Xin Wang, An-Su Wang, Wen-Bo Liao

**Affiliations:** 1 Department of Orthopaedic Surgery, Affiliated Hospital of Zunyi Medical University, Zunyi, 563000 Guizhou China; 2 Joint Orthopaedic Research Center of Zunyi Medical University & University of Rochester Medical Center (JCMR-ZMU & URMC), Zunyi Medical University, Zunyi, 563000 Guizhou China; 3 Institute of Health and Biomedical Innovation, Queensland University of Technology, Brisbane, Queensland 4059 Australia; 4 Translational Research Institute, School of Medicine, University of Queensland, Brisbane, Queensland 4102 Australia

**Keywords:** Endoscopy, Cervical disc herniation, Transcorporeal, Minimally invasive surgery, Discectomy, Channel repair

## Abstract

**Background:**

Compared to anterior cervical discectomy and fusion (ACDF), cervical motion segment and disc was retained through anterior transcorporeal herniotomy (ATH). But surgical field and manipulation in traditional ATH was restricted by the narrow channel. Percutaneous full-endoscopic transdiscal cervical discectomy is a minimally invasive and functional spine surgery. However, significant loss of intervertebral disc height was inevitable. This study was done to illustrate the feasibility, safety, and efficacy and present our surgical experience of percutaneous full-endoscopic anterior transcorporeal cervical discectomy (PEATCD) and channel repair (CR) for the treatment of cervical disc herniation (CDH).

**Methods:**

Four patients with CDH were chosen to undergo PEATCD and CR with a follow-up care for at least 22 months. The visual analogue score (VAS), Japanese Orthopedic Association (JOA), and modified Macnab criteria were recorded during the postoperative periods. CT images were obtained to observe the healing of the channel at 1 week and 3 months after the operation.

**Results:**

The average operating time was 83.75 min. Drainage tubes were unnecessary. No procedure-related complications occurred. The postoperative VAS and JOA scores were improved compared to those of the preoperative assessment. The clinical efficacy was excellent in 3 patients and good in 1 patient at final follow up stage according to the modified Macnab criteria. The hernia was removed completely in all patients according to postoperative MRI. Migration of the repair implementation and collapse of the drilled vertebrae were not observed during the postoperative periods. The bony channel was nearly absent on CT images obtained at 3 months postoperative.

**Conclusion:**

This is the first time that the anterior transcorporeal cervical discectomy and CR have been performed simultaneously under endoscopy. Less damage to disc and the retained cervical motion segment were achieved through this method. This is a feasible, safe, and minimally invasive procedure.

**Trial registration:**

Numbers: ChiCTR1800016383. Registered 29 may 2018. Retrospectively registered. Trial registry: Chinese Clinical Trial Registry.

**Electronic supplementary material:**

The online version of this article (10.1186/s12891-019-2659-0) contains supplementary material, which is available to authorized users.

## Background

Cervical disc herniation (CDH) is a degenerative disease of the cervical spine in which the lesion induces a series of symptoms due to an oppressed spinal cord. Anterior cervical discectomy and fusion (ACDF) has become the standard surgical procedure for single- and multiple-levels degenerative cervical spine diseases due to its excellent clinical results and good fusion rates since it was introduced by Smith and Robinson and then Cloward in the 1950s [1, 2]. However, intervertebral fusion decreases the cervical motion segment and accelerates the degeneration of the adjacent segments [3, 4]. Many other complications are also associated with ACDF [5–8]. Various modifications and procedures have been reported to minimize the surgical disturbance of the biomechanics of the cervical spine [9–15].

With the rapid development of the percutaneous full-endoscopic technique, the endoscopic anterior transdiscal method has become an accepted procedure for CDH [15]. However, significant loss of intervertebral disc height (IDH) in the operated segment has been confirmed in long-term follow-up studies due to the perforating damage to the disc and excessive removal of the nucleus pulposu [15–18]. Anterior transcorporeal discectomy (ATH) was first reported by George in a study that treated lesions in the cervical spinal canal [19], and was derived from a reformation of the technique of Verbiest and Hakuba et al. [20–24]. The transcorporeal technique retains the cervical motion segment, protects the disc from surgical damage and has been modified by several surgeons since its introduction [25–30]. In the 1990s, Nakai sand Sakai improved this procedure by locating the channel close to the center of vertebrae to avoid damage to the longus colli muscle and cervical sympathetic nerve [31, 32]. An operating microscope was used in their procedure to improve surgical vision, cause less damage to soft tissues and increase the safety of the manipulation.

Conventional ATH was performed with a dilator or open approach. The details in the bottom of the channel observed by the naked eye are obscure, even with a microendoscopy, especially if active bleeding is present. Subsequently, the manipulation is difficult for surgeons. Before this study, we conducted percutaneous full-endoscopic anterior transcorporeal cervical discectomy (PEATCD) for patients with central CDH and acquired excellent follow-up outcomes [33]. Due to the merits of the endoscopic system, we greatly reduced the injury to soft tissues and obtained improved vision for the operation. Based on our previous study [33], we performed PEATCD and channel repair (CR) under endoscopy for patients with CDH in order to accelerate the healing process of the bony channel, which has not been reported previously. In this study, a relatively larger bony channel was used compared to our earlier study, which was more beneficial for manipulations and providing sufficient decompression for patients with a large or broad-based hernia. We share our experiences of PEATCD and CR and report the clinical and radiological outcomes during the follow-up periods.

## Materials and methods

### Patient characteristics

This study was approved by local institutional review board (IRB) and the informed consent was acquired from all the patients. In our study, PEATCD with CR was performed for 4 consecutive patients, including 1 female and 3 males with an age range of 41 to 46 years old, with soft CDH from Jul. 2016 to Sep. 2016. The duration of neck pain ranged from 14 to 26 weeks. The demographic characteristics, clinical data, and treatment level are shown in Table 1. The visual analog score (VAS) was used to record the neck pain scores during the postoperative periods. The clinical outcomes were evaluated using the Japanese Orthopedic Association (JOA) scores and modified Macnab criteria (Table 2).

**Table 1:** Summary of demographic characteristics, clinical date, and treatment level

Case	Age (years)	Gender	Level	Duration (weeks)	Postoperative hospital stay (days)	Follow up period (months)
1	43	M	C5–6	14	1	24
2	46	M	C4–5	26	1	24
3	41	M	C4–5	18	1	23
4	46	F	C4–5	17	1	22
Average	44	–	–	18.75	1	23.25

**Table 2:** Modified Macnab Criteria

Grading	Definition
Excellent	Complete resolution of symptoms, recovery of original work activity level and quality of life
good	Mild symptoms, slight activity limitation that do not influence work and quality of life
Fine	Symptoms relieved, activity limitations that influence work and quality of life
bad	No difference in or worsening of symptoms after versus before treatment

### Patient selection

The following were indications for PEATCD and CR. (1) The patient had experienced failure of strict conservative treatment for at least 12 weeks. In our study, 3 patients accepted drug therapy and physical treatment, the left one was treated with epidural ejection; (2) single-level central or mediolateral soft herniation; (3) herniated disc fragment that migrated upward or downward but not free; (4) the symptom or discomfort was caused by the CDH. and (5) there is no evidence of instability in the cervical spine. The contraindications were as follows: (1) Patients with multiple-levels CDH or cervical spinal canal stenosis; (2) posterolateral herniation or foraminal stenosis; (3) previous surgery at the same segment; (4) calcification of the herniated disc or posterior osteophytes of the vertebral body; (5) obesity, (6) the herniation was induced by trauma.

### Endoscopic instruments

The spinal endoscopy system (SPINENDOS GmbH., Munich, Germany) was comprised of a 4.3 mm working channel, an outer sheath with a 6.9 mm diameter, a 30°-angled scope with a continuous water irrigation system, a trephine with a 6.6 mm inner diameter and a 7.6 mm outer diameter, and a low-temperature radiofrequency ablation system (ArthroCare Co., Sunnyvale, CA, USA). The drill was made by NOUVAG AG, Goldach, Switzerland.

### Operative technique

**Fig. 1:**
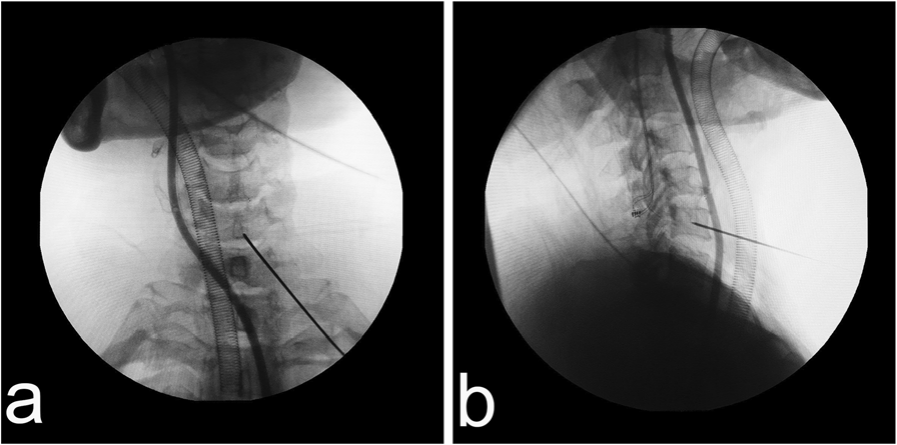
**a**, **b** The position of the K-wire and the outline of the esophagus was confirmed under C-arm fluoroscopy

**Fig. 2:**
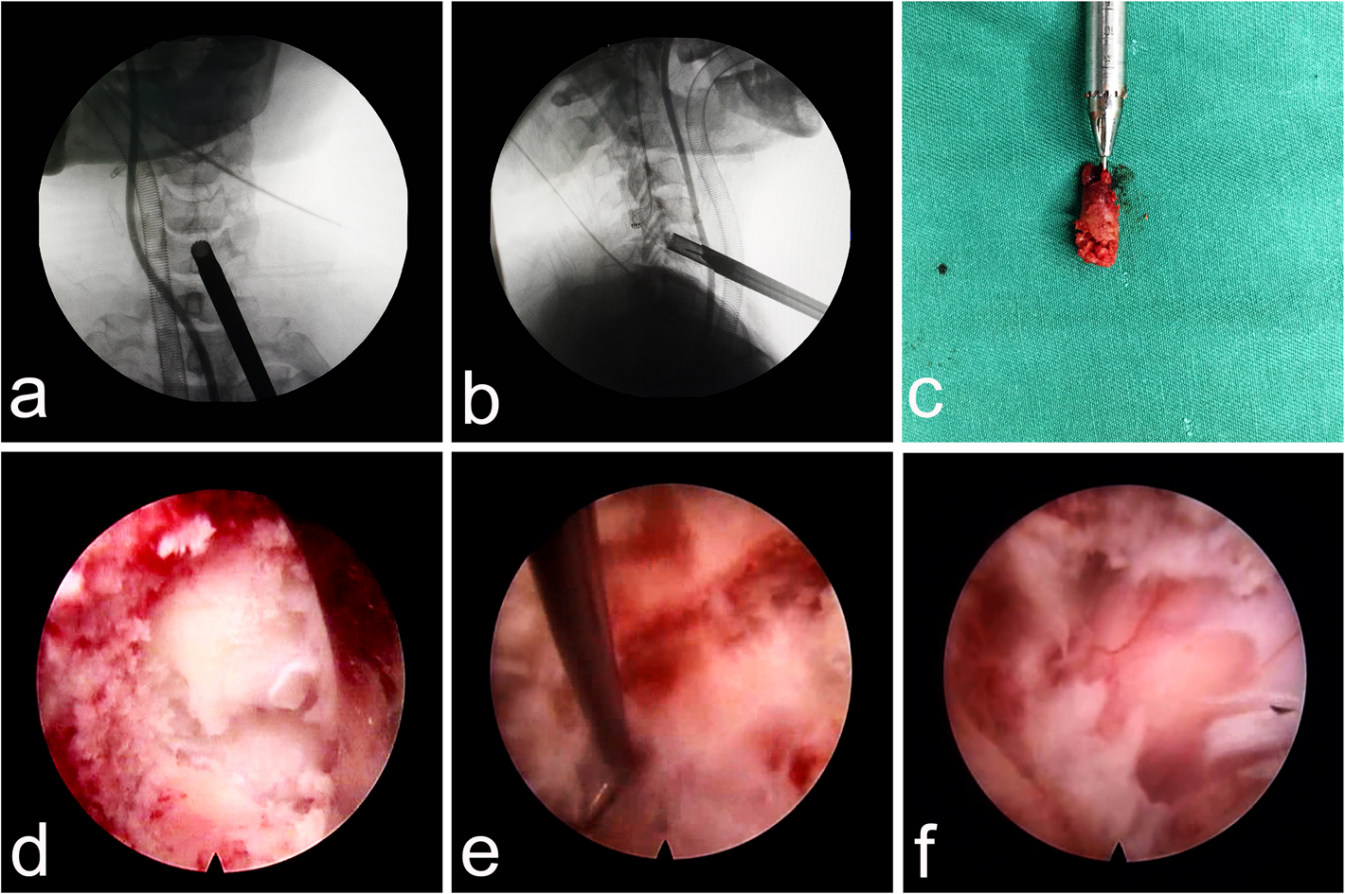
Trephination was terminated when the trephine tip was located at the posterosuperior border of C6 (**a** and **b**). The bone plug (**c**) could be removed by moving the trephine gently in all directions. Some residual bone (**d**) could be seen after inserting the endoscopic system due to the non-parallel plane of the trephine relative to the posterior edge of C6. A hook (**e**) was applied to determine the posterior border of C6, after which the hernia was removed. The dural sac re-expansion (**f**) became apparent when sufficient decompression was achieved

**Fig. 3:**
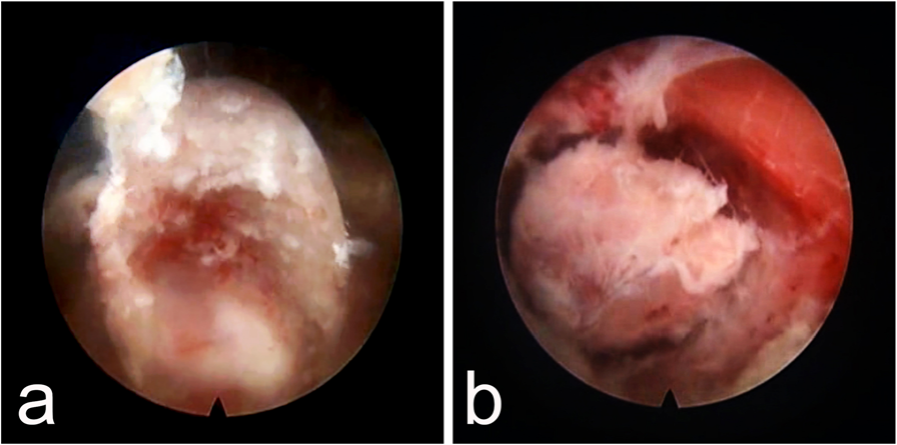
No active bleeding occurred after decompression (**a**), and bone grafting was conducted with the previously harvested bone plug (**b**)

Under general anesthesia, the patient was placed in a supine position with the neck in slight extension. The caudal vertebra was chosen to be drilled for all patients. The entire process was monitored with C-arm fluoroscopy. We used a trephine to directly create the bony channel, which proceeded towards the posterosuperior edge of the targeted vertebra. We primarily confirmed the anterior surface of the drilled vertebra and then selected the channel position with a K-wire under C-arm fluoroscopy (Fig. 1). With the help of two-finger technique (The surgeon located the carotid artery as indicated by its pulsation with the index finger of the left hand and pushed it aside laterally. The tracheoesophagus was then pushed to the medial side with the middle finger), a safe window was created for the insertion of the K-wire and the following operations. The ultrasound examination intraoperative was beneficial if we couldn’t ensure that the vessels had been pushed aside. And the iohexol contrast agent was helpful for confirming the position of the esophagus under C-arm fluoroscopy. We made an approximately 8 mm incision, after which the serial dilators, working cannula and trephine were introduced. We turned the trephine gradually until its tip was located at the posterosuperior border of the vertebra (Fig. 2a and b). Then, the bone plug that was prepared for subsequent CR could be removed together with the trephine by moving it gently in all directions (Fig. 2c). We inserted the working cannula into the bony channel and then inserted the endoscopic system. Bleeding from soft tissues and the channel was resolved by radiofrequency ablation. We could observe some residual bone at the bottom of the channel due to the non-parallel plane between the posterior wall of the vertebra and the bottom of the channel (Fig. 2d). We used a diamond high-speed burr and rongeur to clear these remnants and make a consistent channel towards the herniated lesion. A blunt hook could be used to confirm the posterior wall of the vertebra when a satisfactory channel was established (Fig. 2e). Then, decompression was conducted. To ensure sufficient intraoperative decompression, a small window was made on the posterior longitudinal ligament to directly observe the pulse of the spinal cord (Fig. 2f). When the dural sac re-expansion became apparent, we removed the working cannula from the channel and ensured that no active bleeding was present in the channel. Then, bone grafting was conducted in the channel, in which prior shortening and pruning for the harvested bone had been performed. The insertion process was intently observed via an endoscopy, and the process was terminated when the surfaces of the vertebra and implant were parallel (Fig. 3a and b). We again examined the area for active bleeding before the working cannula and endoscopy were removed. A drainage tube was not necessary. Finally, the residual fluid in the cervical tissues was drained, and the incision was sutured.

### Postoperative and follow-up care

Patients could manage their daily activities 1 day after the operation and could return to general office work 1 week postoperatively. All 4 patients underwent follow-up observation at 1, 3, 6, 12, and 22 months after surgery. At each follow-up stage, the results of the VAS, JOA score, modified Macnab criteria, and neurologic examinations were recorded. MRI was conducted 1 week after the operation to confirm decompression, and CT images were used to observe the bony channel at 1 week and 3 months after the operation.

## Results

**Fig. 4:**
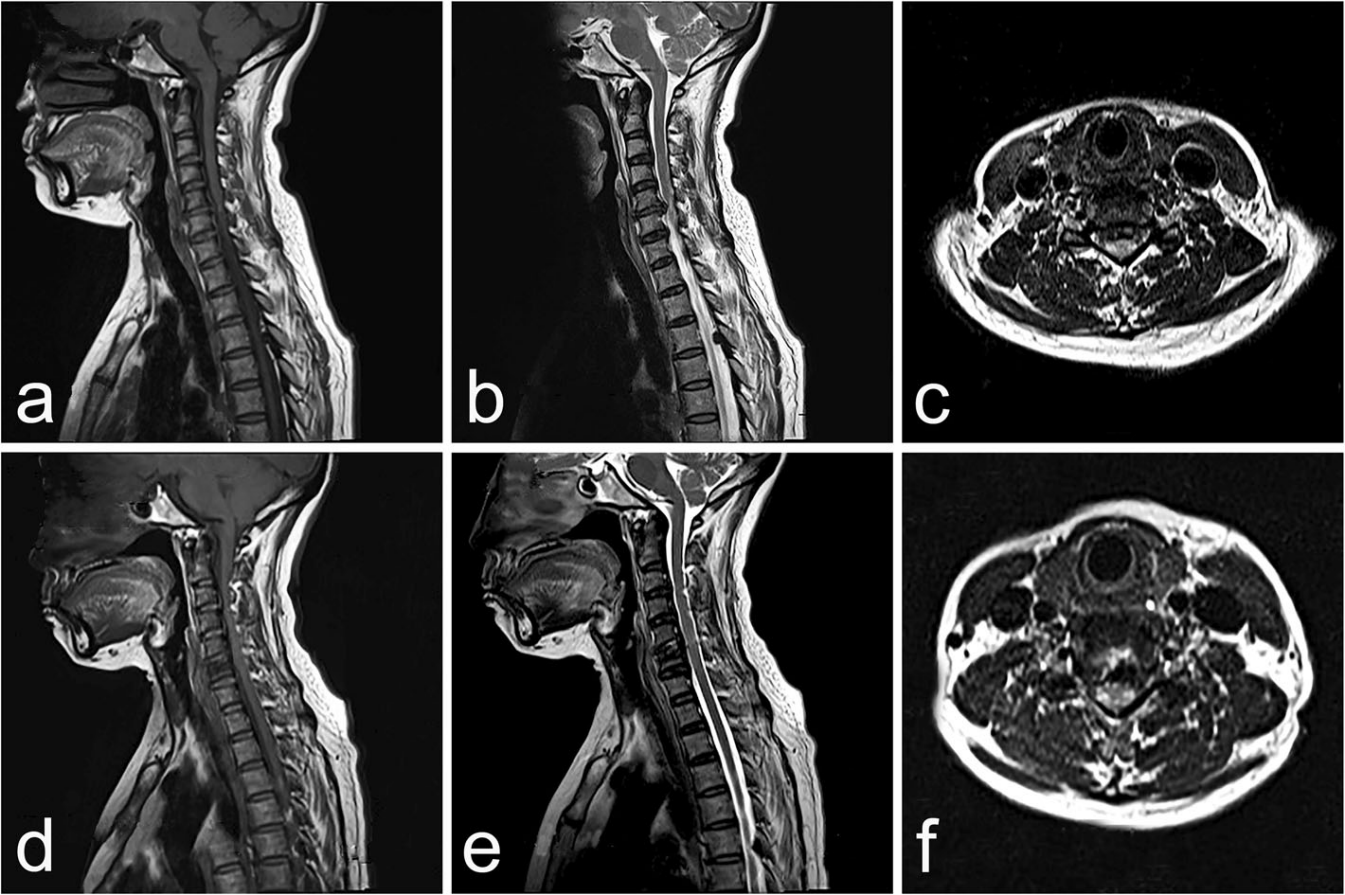
A 43-year-old man was examined due to cervical spondylotic myelopathy. The MRI results showed a broad-based disc hernia at C5/6. The procedure was performed through the channel at C6, and repair was performed with autogenous bone. The postoperative MRI findings showed that the hernia mass was cleared. The preoperative MRI findings, namely, the T1 and T2 weighted sagittal views, are shown (**a** and **b**, respectively). Preoperative MRI, T1 weighted axial view (**c**). Postoperative MRI, T1 and T2 weighted sagittal views (**d** and **e**, respectively). Postoperative MRI, T1 weighted axial view (**f**)

**Fig. 5:**
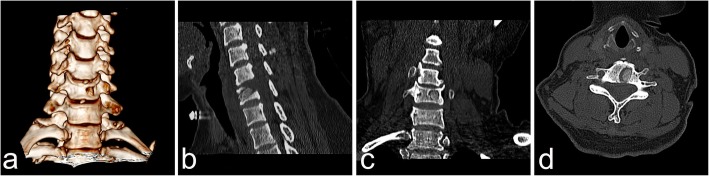
CT images of the channel immediately postoperatively, including the axial plane view (**a**), coronal reconstruction view (**b**), sagittal reconstruction view (**c**), and three-dimensional view (**d**), showed the trajectory of the channel. No migration of the repaired bone occurred

**Fig. 6:**
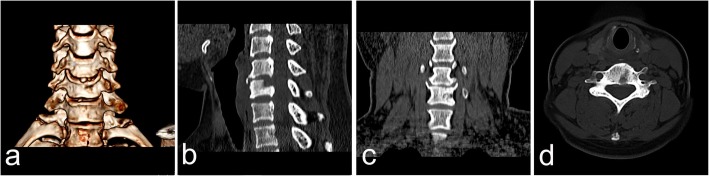
CT images obtained 3 months after the operation, including the axial plane view (**a**), coronal reconstruction view (**b**), sagittal reconstruction view (**c**), and three-dimensional view (**d**), showed that the bone defect had almost completely disappeared. Neither disc space narrowing nor instability were observed

All the procedures were completed successfully by the same surgeon. Three interventions were performed at the C4–5 levels, and 1 intervention was performed at the C5–6 level. The average operation time was 83.75 min (70–105 min). The channel was established at the caudal vertebrae in all patients. Drainage tube was not necessary. The patient in Case 1 exhibited a swollen neck after the operation due to a relatively long operation time. However, no adverse effects were observed, and the tumid neck recovered completely within 2 h. Postoperatively, no manifestations of dysphagia, hematoma, esophageal perforation, or vascular and nerve injury were reported. The postoperative hospital stay was 1 day for the 4 patients. The clinical outcomes of VAS and JOA scores are shown in Table 3. According to the modified Macnab criteria, the clinical efficacy was excellent in 3 cases and good in 1 case after 22 months of follow-up care. Postoperative imaging studies showed that the herniated lesion was completely removed (Fig. 4), and the supplementary files showed the MRI images of the other 3 patients (see Additional file 1). According to the postoperative cervical CT images and radiographs, migration of the repaired bone or collapse of the drilled vertebra did not occur (Fig. 5). The channel disappeared nearly 3 months after the operation (Fig. 6). No instability, kyphosis, or loss of IDH in the cervical spine were observed.

**Table 3:** showed the clinical results of VAS and JOA scores during the postoperative periods

Cases	VAS					JOA			
	Pre	Post-1 m	Post-12 m	Final		Pre	Post-1 m	Post-12 m	Final
1	6	2	0	0		7	12	17	17
2	8	2	0.5	0.5		8	13	15	15.5
3	6	2.5	0	0		7	11	16	16
4	7	3	1	0		9	15	16	16
Average	6.75	2.38	0.38	0.13		7.75	12.75	16	16.13

All the patients got an immediate remission after operation, the pain was disappeared almost at the final follow-up stage, and the average improvement rates of JOA was 90.59%

## Discussion

The superior features of the endoscopic system include the lighting equipment, which supplies sufficient light to the operating area, the magnification system, and continuous irrigation with normal saline. Detailed vision can be acquired in the channel due to these advantages. Continuous irrigation is extremely important. First, the infection risk is further decreased by separating the surgical field from the air. Second, an endoscopic system can ensure relatively improved distinct vision through the hemostasis effect due to hydraulic pressure and exhaustion of blood along with lavage fluid.

Several researchers have previously reported conventional ATH and modified transcorporeal procedures [25, 26, 28–35]. The best points of transcorporeal surgery include preservation of the cervical motion segment and decreasing iatrogenic damage to the pathologic disc. The transcorporeal method had an integrated development that progressed from a traditional open procedure to minimally invasive surgery and finally to the full-endoscopic method. Compared with either ACDF or the transdiscal approach, transcorporeal surgery can better address prolapse of CDH or lesions behind the vertebrae instead of corpectomy in some special situations. In the transcorporeal approach, the damage to the disc depends on the site of the herniated fragments. Injury to the cervical disc will not occur if the hernia deviates from the disc level.

In early procedures, limitations of the channel diameter and surgical vision were inevitable in both the traditional open and microendoscopic ATH. However, ATH under endoscopy can circumvent these limitations. The key point of this novel procedure is the accuracy of the channel trajectory. A diamond high-speed burr was used for channel establishment in most reported studies [31, 32, 35]. In contrast to the early procedures, a trephine was induced to establish the channel in our surgery. The process was dynamically monitored with C-arm fluoroscopy to ensure its depth of entry and the trajectory towards the herniated disc. The channel entrance should be centered relative to the objective drilled vertebra as much as possible to reduce damage to the longus colli muscle, which could reduce not only the subsequent intraoperative bleeding and the risk of postoperative hematoma, but also sympathetic trunk injury due to its anatomical position. The primary purpose of choosing a trephine to drill was to harvest sizable bone for later grafting to the channel. The anatomical integrity was retained, and complications related to bone harvesting from other regions or transplant reaction were avoided. Compared to the diamond high-speed burr, other advantages of using a trephine included better control of the orientation of the channel by the surgeon and a shorter operation time.

In 2010, Lowry combined ACDF with cervical transcorporeal microdecompression and vertebral body access CR for multiple-level CDH [36]. Lowery repaired the channel with a beta-tricalcium phosphate implant filled with locally harvested auto-graft and acquired good results. Although fractures of the drilled vertebrae were not observed, even without CR, in the reported studies [25, 26, 29, 32, 33, 37]. The bony defect was still present 3 months, even 1 year, after transcorporeal procedure in the reported literatures [31, 33]. Whether the risk of collapse will increase has not been reported for cases of patients with osteoporosis or an enlarged channel. In this study, a larger trephine (inner diameter: 6.6 mm, outer diameter: 7.6 mm) was applied to produce the bony channel (approximately 8 mm). Using a larger channel, we could ensure sufficient decompression instead of enlarging the bottom of the channel to obtain a better surgical field, which would make the channel irregular and subsequently increase the risk of repair implant migration. Furthermore, we could address hernias with a broader base (Fig. 4c). CR was conducted while considering the possible collapse of the drilled vertebrae due to the larger channel size. The repair implant was the bone plug harvested intraoperatively with a trephine, which could avoid rejection reaction and greatly decrease the cost. Appropriate pruning to match the harvested bone is necessary before grafting. Additionally, the parallel relationship of the anterior surface of the vertebra and the repair bone must be verified intraoperatively to prevent spinal cord injury due to oppression. Postoperative CT images showed no migration of the repair implementation, and the channel had disappeared almost 3 months after operation and there were no sclerotic changes in the channel. Moreover, no collapse of the drilled vertebrae, loss of IDH, changes of cervical physiological curve, or cervical kyphosis was observed.

Access-related complications in ACDF were avoided with endoscopy because no traction of cervical soft tissues was necessary [7, 8, 38]. Possible severe intraoperative complications included esophageal perforation and vascular injury. A two-finger technique was adopted to pull aside the esophagus and vessels. Additionally, the iohexol contrast agent, which is injected into the gastric tube, could sufficiently delineate the esophageal tract under C-arm fluoroscopy. Subsequently, we could determine whether the esophagus was impaled by observing the relative position of the K-wire and esophagus.

This is the first time anterior transcorporeal cervical discectomy with CR under percutaneous full-endoscopy has been reported. All symptoms of the 4 patients improved during the follow-up periods. Postoperative MRI also showed that the herniated lesion was completely removed. The inclusion criteria for PEATCD and CR was strict, patients with multiple CDH, obesity, or severe myelopathy was excluded. Patient with foraminal stenosis was also excluded because the “Key-hole” technique had been reported safely and effectively in that case [39–41]. Previous study showed that the posterior osteophytes of vertebral body or calcification of the herniated disc fragment was difficult to remove completely [31]. Based on the reported literatures and our limited experiences, patients with central localized soft hernia and without spinal canal stenosis were the ideal candidate for PEATCD and CR [31]. This is a safe and economical novel surgery that offers an alternative for patients with CDH. Additional samples are necessary before widespread application is possible.

## Conclusions

This is the first time that the anterior transcorporeal cervical discectomy with CR have been performed simultaneously under percutaneous full-endoscopy. This novel procedure retains as much anatomical integrity as possible and promotes the healing process of the bony channel by bone grafting. This is a feasible, safe, minimal invasion and novel procedure for patients with CDH.

## Additional file

Additional file 1: Case 2 pre-op cross section, Case 2 pre-op sagittal plane, Case 2 post-op cross section, Case 2 post-op sagittal plane, Case 3 pre-op cross section, Case 3 pre-op sagittal plane, Case 3 post-op cross section, Case 3 post-op sagittal plane, Case 4 pre-op cross section, Case 4 pre-op sagittal plane, Case 4 post-op cross section, and Case 4 post-op sagittal plane. The supplementary figures showed the pre-op and post-op MRI images of the other 3 patients. (ZIP 4930 kb)

## Data Availability

The datasets used during the current study are available from the corresponding author on reasonable request.

## Abbreviations

ACDF: Anterior cervical discectomy and fusion
ATH: Anterior transcorporeal herniotomy
CDH: Cervical disc herniation
CR: Channel repair
IDH: Intervertebral disc height
JOA: Japanese orthopedic association
PEATCD: Percutaneous full-endoscopic anterior transcorporeal cervical discectomy
VAS: Visual analogue score

## References

[1] Cloward RB (1958). The anterior approach for removal of ruptured cervical disks. J Neurosurg.

[2] Smith GW, Robinson RA (1958). The treatment of certain cervical-spine disorders by anterior removal of the intervertebral disc and interbody fusion. J Bone Joint Surg Am.

[3] Hilibrand AS, Carlson GD, Palumbo MA, Jones PK, Bohlman HH (1999). Radiculopathy and myelopathy at segments adjacent to the site of a previous anterior cervical arthrodesis. J Bone Joint Surg Am.

[4] Maiman DJ, Kumaresan S, Yoganandan N, Pintar FA (1999). Biomechanical effect of anterior cervical spine fusion on adjacent segments. Biomed Mater Eng.

[5] Tureyen K (2003). Disc height loss after anterior cervical microdiscectomy with titanium intervertebral cage fusion. Acta Neurochir.

[6] Spanu G, Marchionni M, Adinolfi D, Knerich R (2005). Complications following anterior cervical spine surgery for disc diseases: an analysis of ten years experience. Chir Organi Mov.

[7] Suk KS, Kim KT, Lee SH, Park SW (2006). Prevertebral soft tissue swelling after anterior cervical discectomy and fusion with plate fixation. Int Orthop.

[8] Fountas KN, Kapsalaki EZ, Nikolakakos LG, Smisson HF, Johnston KW, Grigorian AA, Lee GP, Robinson JS (2007). Anterior cervical discectomy and fusion associated complications. Spine.

[9] Dowd GC, Wirth FP (1999). Anterior cervical discectomy: is fusion necessary?. J Neurosurg.

[10] Johnson JP, Filler AG, McBride DQ, Batzdorf U (2000). Anterior cervical foraminotomy for unilateral radicular disease. Spine.

[11] Adamson TE (2001). Microendoscopic posterior cervical laminoforaminotomy for unilateral radiculopathy: results of a new technique in 100 cases. J Neurosurg.

[12] Saringer WF, Reddy B, Nobauer-Huhmann I, Regatschnig R, Reddy M, Tschabitscher M, Knosp E (2003). Endoscopic anterior cervical foraminotomy for unilateral radiculopathy: anatomical morphometric analysis and preliminary clinical experience. J Neurosurg.

[13] Hilton DL (2007). Minimally invasive tubular access for posterior cervical foraminotomy with three-dimensional microscopic visualization and localization with anterior/posterior imaging. Spine J.

[14] Oktenoglu T, Cosar M, Ozer AF, Iplikcioglu C, Sasani M, Canbulat N, Bavbek C, Sarioglu AC (2007). Anterior cervical microdiscectomy with or without fusion. J Spinal Disord Tech.

[15] Ruetten S, Komp M, Merk H, Godolias G (2009). Full-endoscopic anterior decompression versus conventional anterior decompression and fusion in cervical disc herniations. Int Orthop.

[16] Tzaan WC (2011). Anterior percutaneous endoscopic cervical discectomy for cervical intervertebral disc herniation: outcome, complications, and technique. J Spinal Disord Tech.

[17] Lee JH, Lee SH (2014). Clinical and radiographic changes after percutaneous endoscopic cervical discectomy: a long-term follow-up. Photomed Laser Surg.

[18] Yang JS, Chu L, Chen L, Chen F, Ke ZY, Deng ZL (2014). Anterior or posterior approach of full-endoscopic cervical discectomy for cervical intervertebral disc herniation? A comparative cohort study. Spine.

[19] George B, Zerah M, Lot G, Hurth M (1993). Oblique transcorporeal approach to anteriorly located lesions in the cervical spinal canal. Acta Neurochir.

[20] Verbiest H, Paz y Geuse HD (1966). Anterolateral surgery for cervical spondylosis in cases of myelopathy or nerve-root compression. J Neurosurg.

[21] Verbiest H (1968). A lateral approach to the cervical spine: technique and indications. J Neurosurg.

[22] Verbiest H (1973). Chapter 23. The management of cervical spondylosis. Clin Neurosurg.

[23] Verbiest H (1973). Chapter 24. The lateral approach to the cervical spine. Clin Neurosurg.

[24] Hakuba A, Komiyama M, Tsujimoto T, Ahn MS, Nishimura S, Ohta T, Kitano H (1984). Transuncodiscal approach to dumbbell tumors of the cervical spinal canal. J Neurosurg.

[25] Jho HD (1996). Microsurgical anterior cervical foraminotomy for radiculopathy: a new approach to cervical disc herniation. J Neurosurg.

[26] Jho HD, Kim WK, Kim MH (2002). Anterior microforaminotomy for treatment of cervical radiculopathy: part 1--disc-preserving "functional cervical disc surgery**"**. Neurosurgery.

[27] Hong WJ, Kim WK, Park CW, Lee SG, Yoo CJ, Kim YB, Jho HD (2006). Comparison between transuncal approach and upper vertebral transcorporeal approach for unilateral cervical radiculopathy - a preliminary report. Minim Invasive Neurosurg.

[28] Choi G, Lee SH, Bhanot A, Chae YS, Jung B, Lee S (2007). Modified transcorporeal anterior cervical microforaminotomy for cervical radiculopathy: a technical note and early results. Eur Spine J.

[29] Kim JS, Eun SS, Prada N, Choi G, Lee SH (2011). Modified transcorporeal anterior cervical microforaminotomy assisted by O-arm-based navigation: a technical case report. Eur Spine J.

[30] Umebayashi D, Hara M, Nakajima Y, Nishimura Y, Wakabayashi T (2013). Transvertebral anterior cervical foraminotomy: midterm outcomes of clinical and radiological assessments including the finite element method. Eur Spine J.

[31] Nakai S, Yoshizawa H, Kobayashi S, Hayakawa K (2000). Anterior transvertebral herniotomy for cervical disk herniation. J Spinal Disord.

[32] Sakai T, Katoh S, Sairyo K, Tamura T, Hirohashi N, Higashino K, Yasui N (2009). Anterior transvertebral herniotomy for cervical disc herniation: a long-term follow-up study. J Spinal Disord Tech.

[33] Du Q, Wang X, Qin JP, Friis T, Kong WJ, Cai YQ, Ao J, Xu H, Liao WB. Percutaneous full-endoscopic anterior Transcorporeal procedure for cervical disc herniation: a novel procedure and early follow-up study. World Neurosurg. 2017. 10.1016/j.wneu.2017.12.001 29241695

[34] Shim CS, Jung TG, Lee SH (2009). Transcorporeal approach for disc herniation at the C2-C3 level: a technical case report. J Spinal Disord Tech.

[35] Deng ZL, Chu L, Chen L, Yang JS (2016). Anterior transcorporeal approach of percutaneous endoscopic cervical discectomy for disc herniation at the C4-C5 levels: a technical note. Spine J.

[36] Lowry DW, Tuinstra SM, Liang K, Sclafani JA (2015). Clinical outcomes after cervical Transcorporeal microdecompression and vertebral body Access Channel repair. Int J Spine Surg.

[37] Choi G, Arbatti NJ, Modi HN, Prada N, Kim JS, Kim HJ, Myung SH, Lee SH (2010). Transcorporeal tunnel approach for unilateral cervical radiculopathy: a 2-year follow-up review and results. Minim Invasive Neurosurg.

[38] Traynelis VC, Malone HR, Smith ZA, Hsu WK, Kanter AS, Qureshi SA, Cho SK, Baird EO, Isaacs RE, Rahman RK (2017). Rare complications of cervical spine surgery: Horner's syndrome. Global Spine J.

[39] Ahn J, Tabaraee E, Bohl DD, Singh K (2015). Minimally invasive posterior cervical Foraminotomy. J Spinal Disord Tech.

[40] Kim CH, Shin KH, Chung CK, Park SB, Kim JH (2015). Changes in cervical sagittal alignment after single-level posterior percutaneous endoscopic cervical diskectomy. Global Spine J.

[41] Zhang C, Wu J, Xu C, Zheng W, Pan Y, Li C, Zhou Y (2018). Minimally invasive full-endoscopic posterior cervical Foraminotomy assisted by O-arm-based navigation. Pain Physician.

