# Supplementary figures and images for: Percutaneous full-endoscopic anterior transcorporeal cervical discectomy and channel repair: a technique note report
Source: BMC Musculoskelet Disord. 2019 Jun 10;20:280. doi: 10.1186/s12891-019-2659-0 (PMC6558825; doi:10.1186/s12891-019-2659-0)

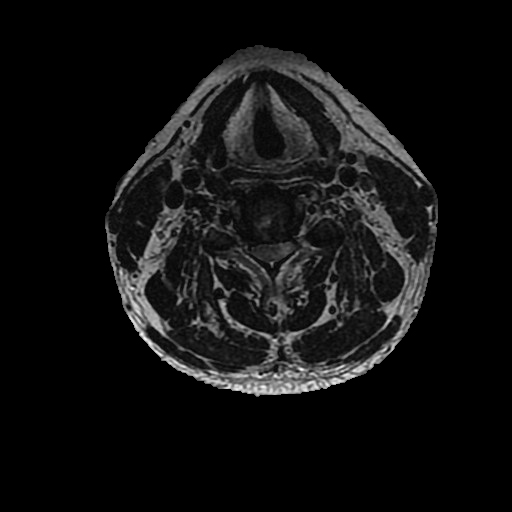

Supplement: Supplementary file 1 — Case 2 pre-op cross section, Case 2 pre-op sagittal plane, Case 2 post-op cross section, Case 2 post-op sagittal plane, Case 3 pre-op cross section, Case 3 pre-op sagittal plane, Case 3 post-op cross section, Case 3 post-op sagittal plane, Case 4 pre-op cross section, Case 4 pre-op sagittal plane, Case 4 post-op cross section, and Case 4 post-op sagittal plane. The supplementary figures showed the pre-op and post-op MRI images of the other 3 patients. (ZIP 4930 kb) [file 12891_2019_2659_MOESM1_ESM.zip › Case 2 Post-op Cross SectionR3.jpg]

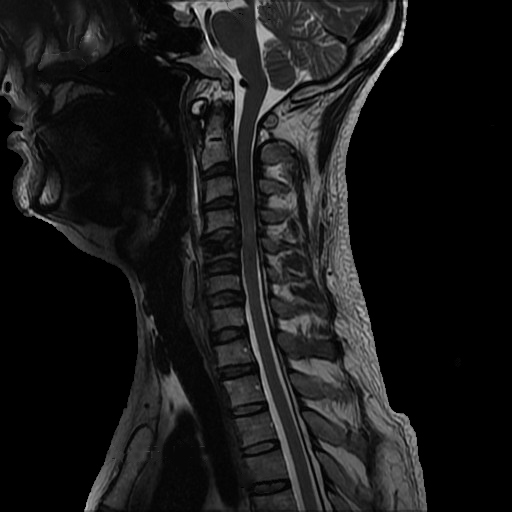

Supplement: Supplementary file 1 — Case 2 pre-op cross section, Case 2 pre-op sagittal plane, Case 2 post-op cross section, Case 2 post-op sagittal plane, Case 3 pre-op cross section, Case 3 pre-op sagittal plane, Case 3 post-op cross section, Case 3 post-op sagittal plane, Case 4 pre-op cross section, Case 4 pre-op sagittal plane, Case 4 post-op cross section, and Case 4 post-op sagittal plane. The supplementary figures showed the pre-op and post-op MRI images of the other 3 patients. (ZIP 4930 kb) [file 12891_2019_2659_MOESM1_ESM.zip › Case 2 Post-op Sagittal PlaneR3.jpg]

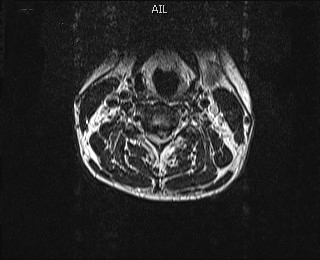

Supplement: Supplementary file 1 — Case 2 pre-op cross section, Case 2 pre-op sagittal plane, Case 2 post-op cross section, Case 2 post-op sagittal plane, Case 3 pre-op cross section, Case 3 pre-op sagittal plane, Case 3 post-op cross section, Case 3 post-op sagittal plane, Case 4 pre-op cross section, Case 4 pre-op sagittal plane, Case 4 post-op cross section, and Case 4 post-op sagittal plane. The supplementary figures showed the pre-op and post-op MRI images of the other 3 patients. (ZIP 4930 kb) [file 12891_2019_2659_MOESM1_ESM.zip › Case 2 Pre-op Cross SectionR3.jpg]

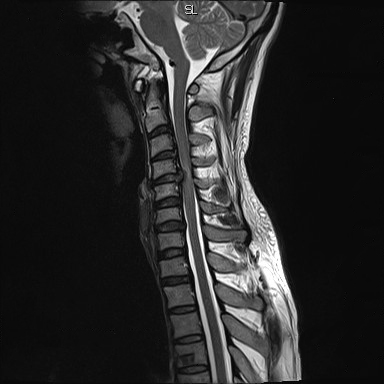

Supplement: Supplementary file 1 — Case 2 pre-op cross section, Case 2 pre-op sagittal plane, Case 2 post-op cross section, Case 2 post-op sagittal plane, Case 3 pre-op cross section, Case 3 pre-op sagittal plane, Case 3 post-op cross section, Case 3 post-op sagittal plane, Case 4 pre-op cross section, Case 4 pre-op sagittal plane, Case 4 post-op cross section, and Case 4 post-op sagittal plane. The supplementary figures showed the pre-op and post-op MRI images of the other 3 patients. (ZIP 4930 kb) [file 12891_2019_2659_MOESM1_ESM.zip › Case 2 Pre-op Sagittal PlaneR3.jpg]

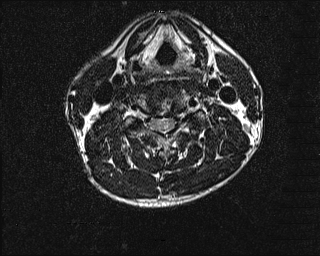

Supplement: Supplementary file 1 — Case 2 pre-op cross section, Case 2 pre-op sagittal plane, Case 2 post-op cross section, Case 2 post-op sagittal plane, Case 3 pre-op cross section, Case 3 pre-op sagittal plane, Case 3 post-op cross section, Case 3 post-op sagittal plane, Case 4 pre-op cross section, Case 4 pre-op sagittal plane, Case 4 post-op cross section, and Case 4 post-op sagittal plane. The supplementary figures showed the pre-op and post-op MRI images of the other 3 patients. (ZIP 4930 kb) [file 12891_2019_2659_MOESM1_ESM.zip › Case 3 Post-op Cross SectionR3.jpg]

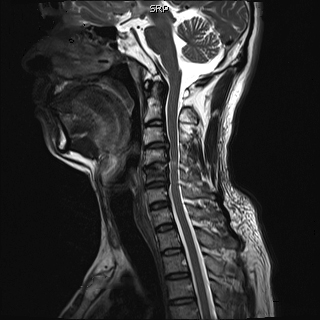

Supplement: Supplementary file 1 — Case 2 pre-op cross section, Case 2 pre-op sagittal plane, Case 2 post-op cross section, Case 2 post-op sagittal plane, Case 3 pre-op cross section, Case 3 pre-op sagittal plane, Case 3 post-op cross section, Case 3 post-op sagittal plane, Case 4 pre-op cross section, Case 4 pre-op sagittal plane, Case 4 post-op cross section, and Case 4 post-op sagittal plane. The supplementary figures showed the pre-op and post-op MRI images of the other 3 patients. (ZIP 4930 kb) [file 12891_2019_2659_MOESM1_ESM.zip › Case 3 Post-op Sagittal PlaneR3.jpg]

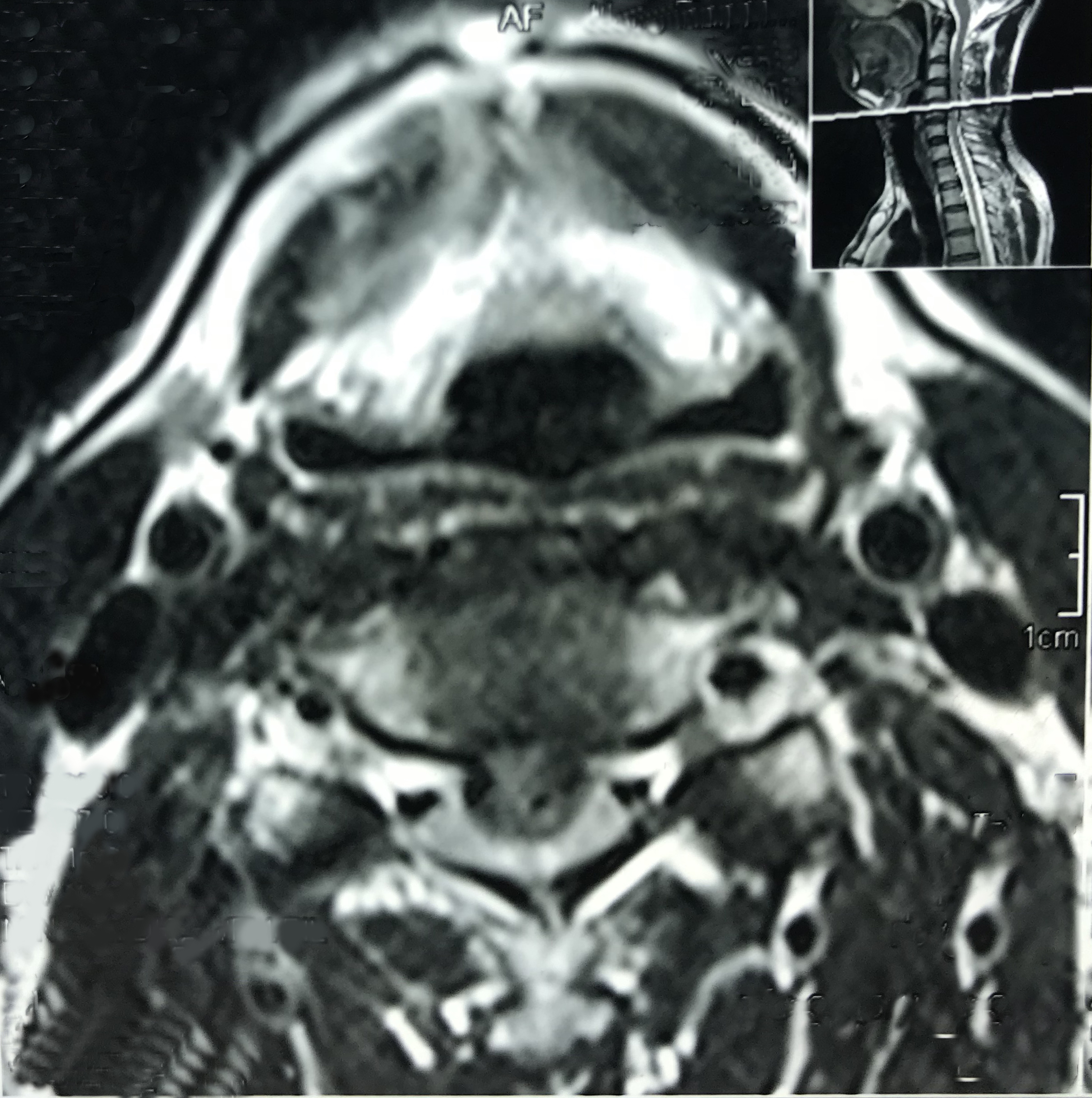

Supplement: Supplementary file 1 — Case 2 pre-op cross section, Case 2 pre-op sagittal plane, Case 2 post-op cross section, Case 2 post-op sagittal plane, Case 3 pre-op cross section, Case 3 pre-op sagittal plane, Case 3 post-op cross section, Case 3 post-op sagittal plane, Case 4 pre-op cross section, Case 4 pre-op sagittal plane, Case 4 post-op cross section, and Case 4 post-op sagittal plane. The supplementary figures showed the pre-op and post-op MRI images of the other 3 patients. (ZIP 4930 kb) [file 12891_2019_2659_MOESM1_ESM.zip › Case 3 Pre-op Cross SectionR3.JPG]

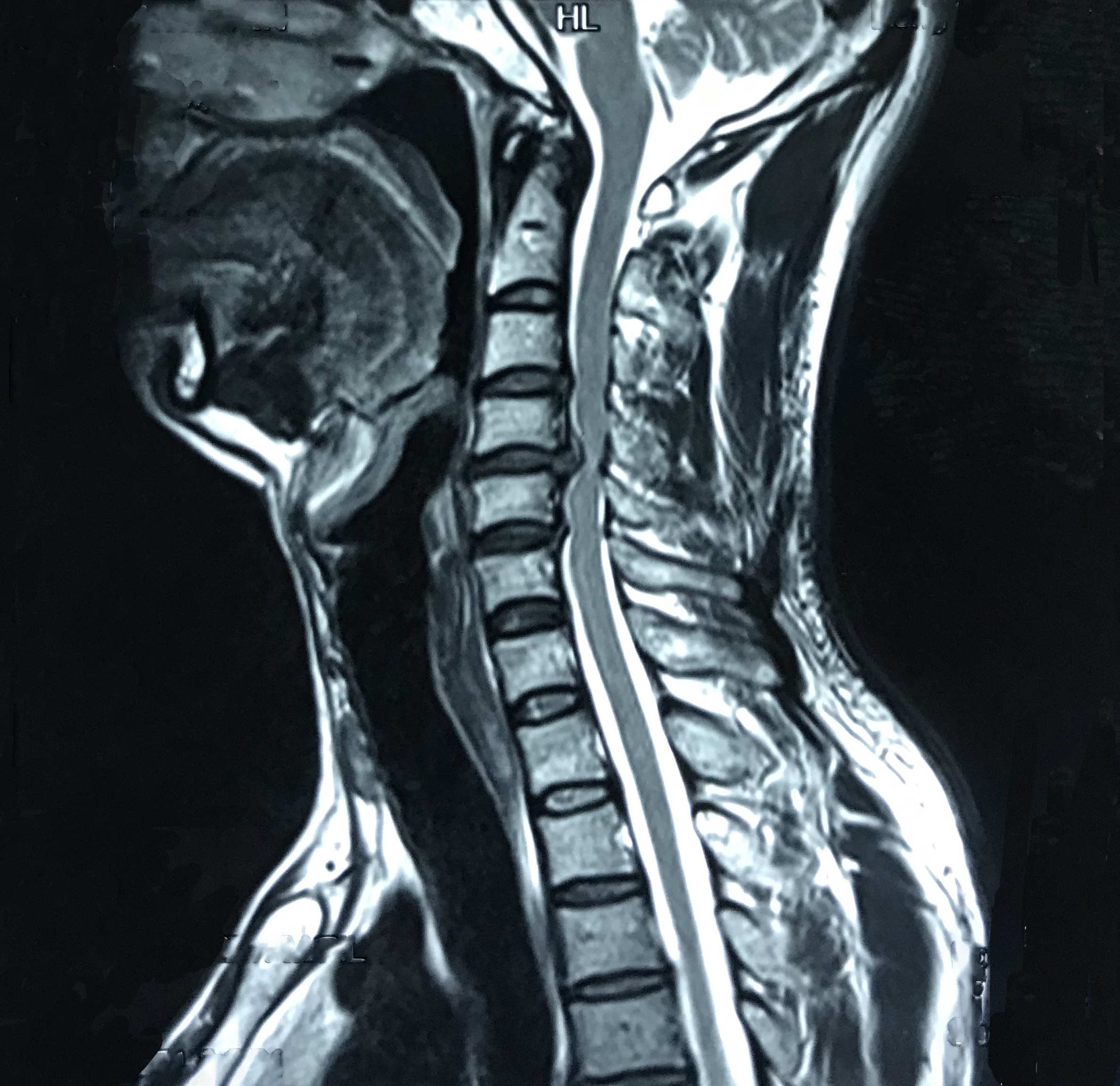

Supplement: Supplementary file 1 — Case 2 pre-op cross section, Case 2 pre-op sagittal plane, Case 2 post-op cross section, Case 2 post-op sagittal plane, Case 3 pre-op cross section, Case 3 pre-op sagittal plane, Case 3 post-op cross section, Case 3 post-op sagittal plane, Case 4 pre-op cross section, Case 4 pre-op sagittal plane, Case 4 post-op cross section, and Case 4 post-op sagittal plane. The supplementary figures showed the pre-op and post-op MRI images of the other 3 patients. (ZIP 4930 kb) [file 12891_2019_2659_MOESM1_ESM.zip › Case 3 Pre-op Sagittal PlaneR3.JPG]

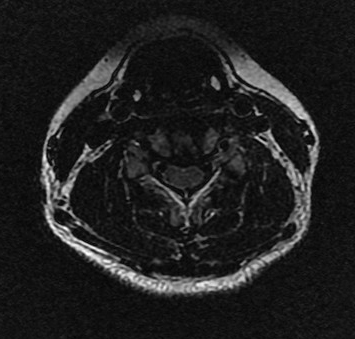

Supplement: Supplementary file 1 — Case 2 pre-op cross section, Case 2 pre-op sagittal plane, Case 2 post-op cross section, Case 2 post-op sagittal plane, Case 3 pre-op cross section, Case 3 pre-op sagittal plane, Case 3 post-op cross section, Case 3 post-op sagittal plane, Case 4 pre-op cross section, Case 4 pre-op sagittal plane, Case 4 post-op cross section, and Case 4 post-op sagittal plane. The supplementary figures showed the pre-op and post-op MRI images of the other 3 patients. (ZIP 4930 kb) [file 12891_2019_2659_MOESM1_ESM.zip › Case 4 Post-op Cross SectionR3.jpg]

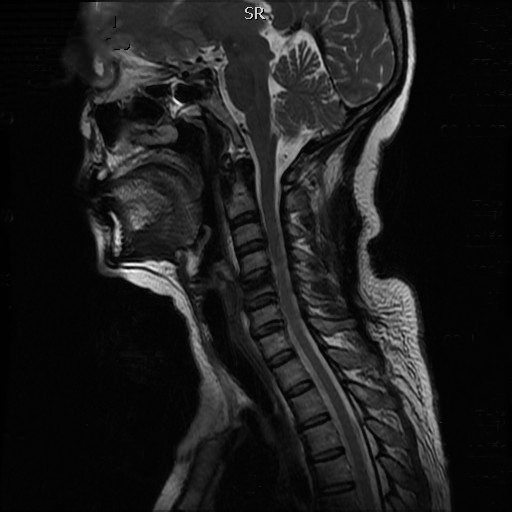

Supplement: Supplementary file 1 — Case 2 pre-op cross section, Case 2 pre-op sagittal plane, Case 2 post-op cross section, Case 2 post-op sagittal plane, Case 3 pre-op cross section, Case 3 pre-op sagittal plane, Case 3 post-op cross section, Case 3 post-op sagittal plane, Case 4 pre-op cross section, Case 4 pre-op sagittal plane, Case 4 post-op cross section, and Case 4 post-op sagittal plane. The supplementary figures showed the pre-op and post-op MRI images of the other 3 patients. (ZIP 4930 kb) [file 12891_2019_2659_MOESM1_ESM.zip › Case 4 Post-op Sagittal PlaneR3.jpg]

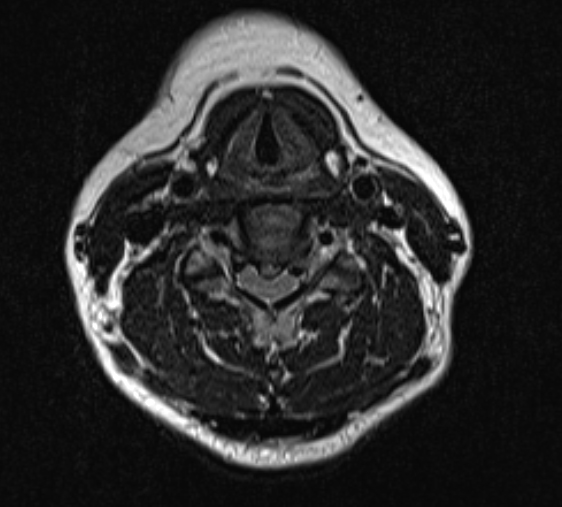

Supplement: Supplementary file 1 — Case 2 pre-op cross section, Case 2 pre-op sagittal plane, Case 2 post-op cross section, Case 2 post-op sagittal plane, Case 3 pre-op cross section, Case 3 pre-op sagittal plane, Case 3 post-op cross section, Case 3 post-op sagittal plane, Case 4 pre-op cross section, Case 4 pre-op sagittal plane, Case 4 post-op cross section, and Case 4 post-op sagittal plane. The supplementary figures showed the pre-op and post-op MRI images of the other 3 patients. (ZIP 4930 kb) [file 12891_2019_2659_MOESM1_ESM.zip › Case 4 Pre-op Cross SectionR3.jpg]

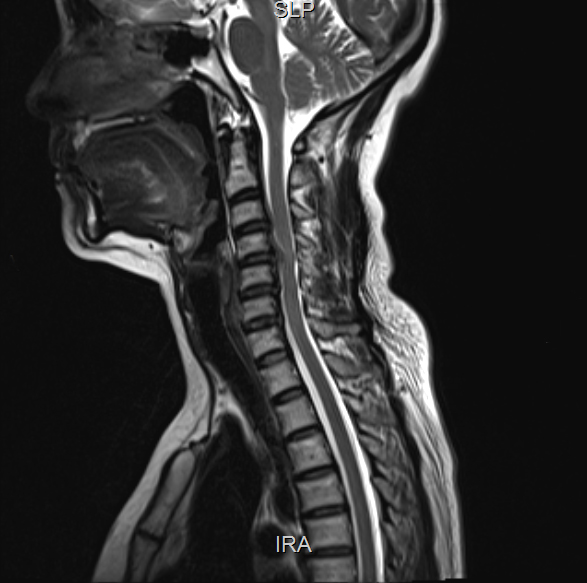

Supplement: Supplementary file 1 — Case 2 pre-op cross section, Case 2 pre-op sagittal plane, Case 2 post-op cross section, Case 2 post-op sagittal plane, Case 3 pre-op cross section, Case 3 pre-op sagittal plane, Case 3 post-op cross section, Case 3 post-op sagittal plane, Case 4 pre-op cross section, Case 4 pre-op sagittal plane, Case 4 post-op cross section, and Case 4 post-op sagittal plane. The supplementary figures showed the pre-op and post-op MRI images of the other 3 patients. (ZIP 4930 kb) [file 12891_2019_2659_MOESM1_ESM.zip › Case 4 Pre-op Sagittal PlaneR3.jpg]
